# Citation Advantage of Promoted Articles in a Cross-Publisher Distribution Platform: 36-Month Follow-up to a Randomized Controlled Trial

**DOI:** 10.2196/34051

**Published:** 2021-12-10

**Authors:** Paul Kudlow, Tashauna Brown, Gunther Eysenbach

**Affiliations:** 1 Department of Psychiatry University of Toronto Toronto, ON Canada; 2 TrendMD Inc Toronto, ON Canada; 3 University of Victoria Victoria, BC Canada

**Keywords:** knowledge translation, knowledge, dissemination, digital knowledge translation, digital publishing, e-publishing, open access, scientometrics, infometrics

## Abstract

**Background:**

There are limited evidence-based strategies that have been shown to increase the rate at which peer-reviewed articles are cited. In a previously reported randomized controlled trial, we demonstrated that promotion of article links in an online cross-publisher distribution platform (TrendMD) persistently augments citation rates after 12 months, leading to a statistically significant 50% increase in citations relative to the control.

**Objective:**

This study aims to investigate if the citation advantage of promoted articles upholds after 36 months.

**Methods:**

A total of 3200 published articles in 64 peer-reviewed journals across 8 subject areas were block randomized at the subject level to either the TrendMD group (n=1600) or the control group (n=1600) of the study. Articles were promoted in the TrendMD Network for 6 months. We compared the citation rates in both groups after 36 months.

**Results:**

At 36 months, we found the citation advantage endured; articles randomized to TrendMD showed a 28% increase in mean citations relative to the control. The difference in mean citations at 36 months for articles randomized to TrendMD versus the control was 10.52 (95% CI 3.79-17.25) and was statistically significant (*P*=.001).

**Conclusions:**

To our knowledge, this is the first randomized controlled trial to demonstrate how a postpublication article promotion intervention can be used to persistently augment citations of peer-reviewed articles. TrendMD is an efficient digital tool for knowledge translation and dissemination to targeted audiences to facilitate the uptake of research.

## Introduction

Citations are a leading indicator of scholarly impact. They measure the spread of new knowledge; acknowledge the contribution of colleagues; and, in many fields, form the basis of tenure and promotion or even direct compensation [1,2]. Citations accrue from research getting noticed and used by authors when creating their own scholarly work. The problem is, there is a growing mismatch between the number of newly published articles and the number of papers an academic can discover and ultimately cite in the creation of their own scholarly work. There are over 8000 new peer-reviewed articles published daily, a figure that is growing exponentially, making it challenging for scholars to sift through potentially relevant literature [3,4]. As a result, many relevant papers that could be cited are missed by scholars [3]. Accordingly, data suggests that roughly 35% of articles published between 1990 and 2015 remain uncited; a figure that may be increasing [5]. A study published in 2021 found that the increasing enormity of academic literature may be impeding on the rise, progress, and adoption of new ideas; the authors suggest that changes are needed in scholarship dissemination to increase the discoverability of new concepts that researchers may not be searching for [6]. The growing mismatch between the number of articles published and the ability for readers to come across articles that they were not already seeking out has created a pressing need for strategies that augment the discoverability of scholarly content and to target content to potential knowledge users. Postpublication strategies such as promotion of articles in social media and other content distribution networks promise to enhance discoverability, which may increase the chances that the most relevant paper is found and ultimately cited by scholars in the creation of their own work. However, how to target promotional campaigns to the right audience remains an open question, and there is currently limited evidence showing which strategies yield a positive effect on scholarly article impact, such as that reflected by citation counts.

One of the most widely studied strategies examined to increase discoverability, accessibility, and consequential citation counts of scholarly work is publishing content in open access (OA) journals. OA increases visibility and hence can have a positive effect on usage metrics such as the number of downloads [7], but the effect on citations is less clear and likely discipline dependent [8-12]. Since the first carefully designed multivariate observational study in 2006 showed a clear OA citation advantage even when adjusted for possible confounders [8,9,13], several large observational studies have found OA articles accrue between 50% to 200% more citations compared to closed access articles; this is referred to as the “open access citation advantage” (OACA) [8,14]. The OACA, however, has not been universally accepted [7,15,16]. For example, in 2011, Davis [16] completed a randomized controlled trial study showing that making articles free and open did not yield an increase in citation counts over a 3-year period relative to closed access articles. Part of the reason why the OACA has been found in observational data and not experimental data is likely due to residual confounders. Even though the original study suggesting an OA citation advantage was carefully adjusted for multiple confounders [8], selection bias cannot be fully adjusted for and is one of the biggest confounders identified in observational research examining the OACA [17]; more prominent authors are more likely to pay to publish OA articles, and if authors are more likely to provide access to their “highest quality” articles, then OA articles will have more citations than closed access articles [11,18]. Furthermore, as OA content becomes increasingly ubiquitous in the scholarly ecosystem, the OACA, if any, is likely to extinguish, as all articles share the same OA characteristic.

Aside from publishing research in an OA journal, other studies have suggested that the promotion of articles in social media platforms may be used to augment the page views and citations of articles, but this too remains highly controversial. For example, two recent studies [19,20] found that intensive social media promotion significantly increased page views of scholarly articles. A more recent study [21] found that an intensive social media promotion strategy yielded a citation advantage to promoted articles. However, the findings of the Luc et al [21] study have come under considerable scrutiny due to significant methodological flaws that suggests that social media promotion did not yield a citation benefit at all [22-24]. The most significant methodological flaw was the fact that the papers listed in the Luc et al [21] paper may have not matched those described by their methods. Similarly, two rigorous randomized controlled trials [25,26] found that the promotion of articles in social media did not yield any increase in article page views. These results refer to organic tweeting, and an unexamined question remains if promoted tweets, which measurably lead to more visits, also lead to more citations. Taken together, though there are mixed data as to whether social media promotion increases article page views, there are currently no replicated data to suggest that the strategy yields a citation advantage to promoted articles.

Our group previously published studies examining the extent to which the promotion of articles in a novel cross-publisher distribution platform (TrendMD) increases article page views, usage, and citation counts. In a 2014 study, TrendMD article promotion led to an 87% increase in page views relative to the control in a 4-week randomized controlled trial [27]. These data were replicated in in a 3-week crossover trial that found that promotion of articles in the TrendMD Network yielded a 30% and 49% weekly increase in page views relative to the control [28]. In 2017, we completed a 4-week randomized controlled trial that found articles randomized to TrendMD had a 77% increase in article saves on Mendeley relative to the control [29]. These findings were particularly significant because Mendeley saves are not only a robust measure of article usage, but the metric is also strongly correlated to future citations [30-34]. Building on these data, in 2019, we conducted a randomized controlled trial including 3200 articles published across 8 subject areas (eg, medicine, physics, business, and humanities) and found that promotion of articles over 6 months conferred an overall statistically significant 50% citation advantage to promoted articles relative to the control at 12 months [35]. TrendMD promotion increased citations for 3 of 8 disciplines tested, yielding the largest citation advantage to articles published in the subject areas of health, medical, and life sciences. Taken together, results of our studies suggest that TrendMD confers a short-term page view, usage, and citation advantage; however, we do not know whether the measured advantages persist over time or becomes diluted by other factors [36].

In this study, we conducted a follow-up analysis to our 12-month randomized controlled trial to determine whether the citation advantage persists at 36 months. We also sought to determine whether TrendMD’s effect on citation counts were specific to particular disciplines or consistent across all disciplines. We hypothesized that the promotion of articles in TrendMD would yield a persistent citation advantage at 36 months. We further hypothesized that this effect would be seen across more disciplines than initially measured at 12 months.

## Methods

### Summary

The majority of the Methods section included herein were copied verbatim from the Methods section we describe in our previously published 12-month randomized controlled trial [35].

We conducted a 36-month randomized controlled trial that included 3200 articles published in 64 peer-reviewed journals across 8 subject areas. The length of the study was 6 months for the intervention and an additional 30 months of observation for a total of 36 months. We published our initial findings at 12 months [35]. We measured citations at 6, 12, and 36 months. The subject areas/categories were selected based on the 8 categories listed in Google Scholar [37]. The categories selected were business economics and management; chemical and materials sciences; engineering and computer science; health and medical sciences; humanities, literature, and arts; life sciences and earth sciences; physics and mathematics; and social sciences. For each subject area, the top 20 journals ranked by Google Scholar’s h-5 index were selected (Google Scholar only displays the top 20 journals in each subject area). Please see the supplementary material published in our 2019 study [35] for our rationale for using Google Scholar and the h-5 index in our selection criteria. Eight journals were then randomly selected with a random number generator from the top 20 in each subject area to be included in the study; we did this as opposed to selecting the top journals in each subject area so that our sample would include a randomized mixture of journals of high and lower impact in each subject area. Including both high and lower impact journals was important to our study because we wanted to mitigate the potential confounder that TrendMD promotion is only effective in high impact journals. Journals that were not indexed in Scopus or Web of Science were excluded from the study. Preprint servers such as ArXiv were also excluded from the study.

**Figure 1:**
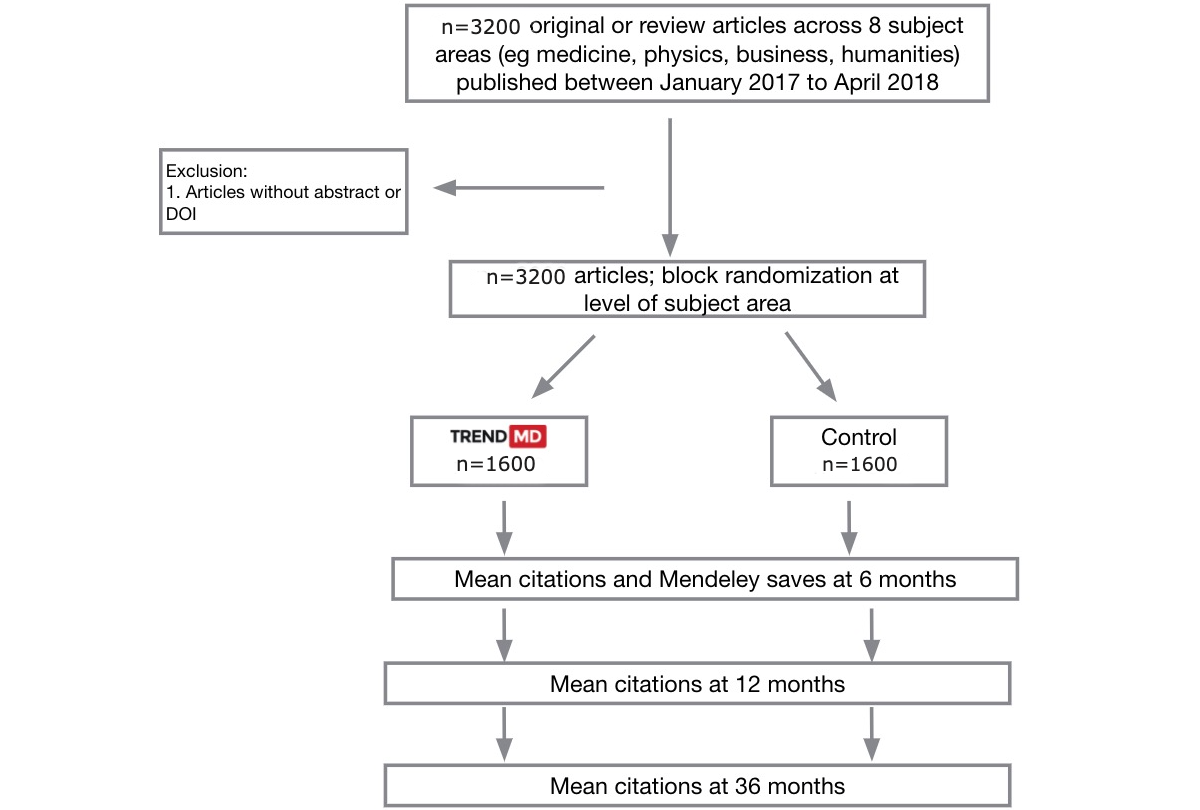
Overall study design.

Starting from April 2018, 50 of the most recently published original articles or review articles in each journal were selected for inclusion in the study. Articles selected for inclusion were published online between January 2017 and April 2018; this includes early view articles. Articles were excluded if they did not contain an abstract or DOI. Block randomization using a random number generator at the subject level was used to randomize articles to either the control or the intervention arm of the study. For each subject area, 200 articles were randomized to the control, and 200 articles were randomized to the intervention. In total, 1600 articles were randomized to the control, and 1600 articles were randomized to the intervention. The overall study design is presented in Figure 1.

### Intervention

**Figure 2:**
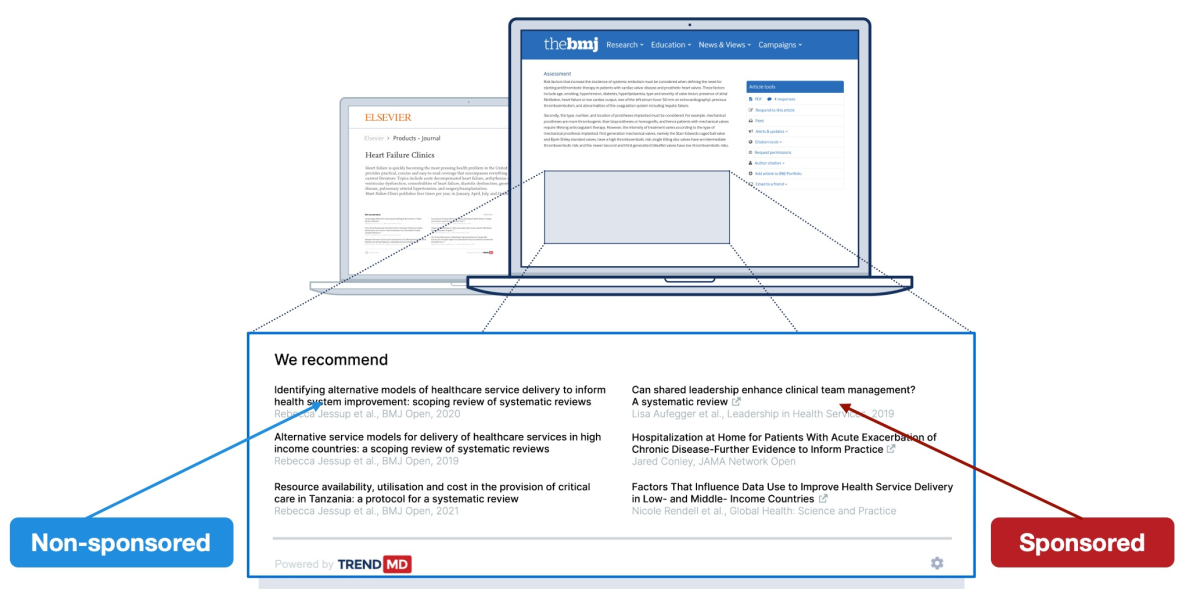
TrendMD: sponsored versus nonsponsored links.

**Figure 3:**
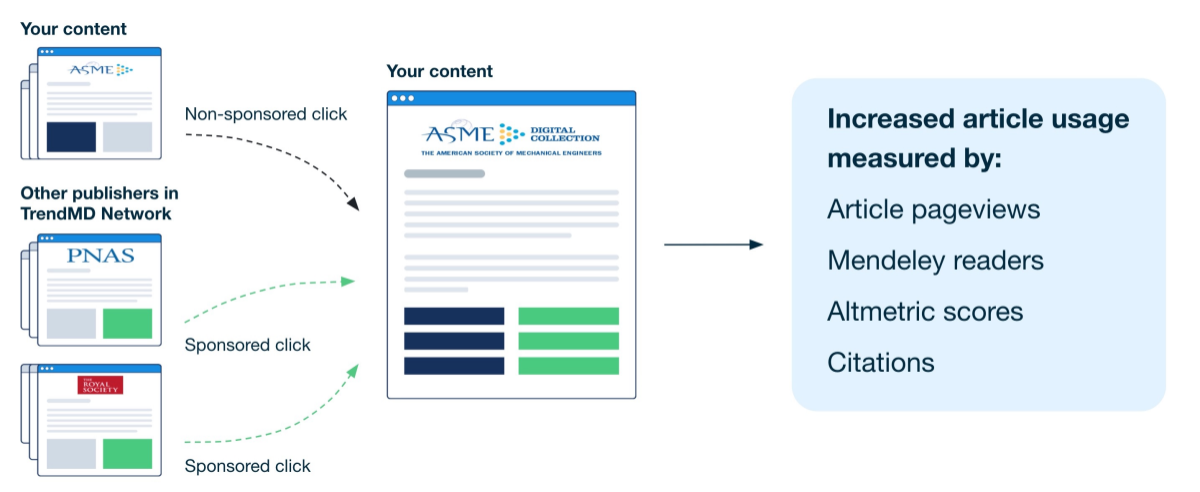
How the TrendMD Network works.

**Figure 4:**
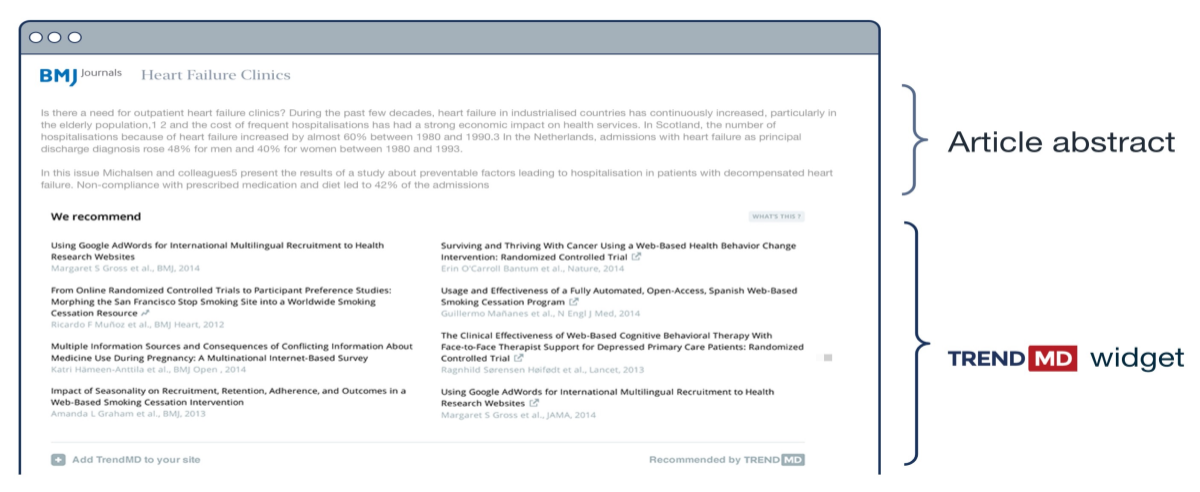
TrendMD widget display.

TrendMD [38] is a cross-publisher article recommendation and distribution platform that, as of May 2019, was embedded on over 4700 journals and websites from 300 publishers and seen by approximately 125 million readers per month. Roughly two-thirds of the TrendMD Network is related to scientific, technical, and medical (STM) publications; the other one-third is a relatively even split between social sciences, humanities, and business publications. Participating publishers use TrendMD to distribute their published article links within the article recommendations displayed on articles within their journals (nonsponsored recommendations) or third-party journals within the TrendMD Network (sponsored recommendations; please see Figures 2 and 3). TrendMD’s content distribution model is benchmarked to similar services in the consumer web, where the leading networks Outbrain [39] and Taboola [40] generate the “From the web” and “You may like” recommendations seen alongside the content on many popular websites like CNN or BBC [29,35] (please see Figure 4 for reference).

The intervention consisted of the promotion of 1600 articles in the TrendMD Network for 6 months, between May 1, 2018, and November 2, 2018. Articles included in the TrendMD Network are displayed as recommended article links. Links to articles randomized to TrendMD were displayed as sponsored recommended links on publications participating in the TrendMD Network. There was an average of 4300 participating journals and 121 million readers per month during the course of the study. The frequency of sponsored article link placements is determined by a relevancy score based on the following: relatedness (ie, keyword overlap), collaborative filtering (similar to Amazon’s “people who bought this item also bought that item”), and user clickstream analysis (the Netflix approach, basing recommendations on the users’ interests expressed through their online history) [27-29,35]. As a result of the relevancy scoring system, some articles randomized to TrendMD were both seen more often (ie, accrued more link impressions) and clicked on more frequently than others in the TrendMD Network.

The sponsored links are displayed in the TrendMD Network as long as they are relevant and the advertiser account balance is greater than US $0. An account in TrendMD was created for this study. The 1600 articles randomized to TrendMD received a maximum total budget of US $9600 at a cost-per-click of US $0.10 for 96,000 sponsored TrendMD clicks. The account was allowed to spend up to a maximum for US $1600 per month, or 16,000 clicks per month, over the 6-month study period for a total of 96,000 clicks. The actual amount spent by the account was US $1600 per month; all clicks were delivered each month throughout the 6-month study. A summary of how TrendMD works and the outcomes measured can be seen in Figure 3.

### Control

Articles randomized to the control (n=1600) received no promotion in the TrendMD Network. Articles randomized to the control received traffic by organic means (eg, Google, Google Scholar, or PubMed) and other means implemented by publishers or authors of content outside the context of this study.

### Primary Outcome

The primary outcome of our study is the mean citation counts for articles randomized to TrendMD compared to the control at 36 months. Article citation counts at 36 months were extracted through the Scopus application programming interface (API) on March 30, 2021.

### Secondary Outcomes

#### Thirty-six–Month Analysis

Mean citation counts after 36 months were compared for articles randomized to TrendMD versus control for each of the 8 subject areas. This analysis was completed to assess whether the effects of TrendMD promotion were discipline specific. Article citation counts were extracted from the Scopus API on March 30, 2021.

#### Twelve- and Six-Month Analysis

Mean citation counts after 12 and 6 months were compared for articles randomized to TrendMD versus the control. Article citation counts were extracted from the Scopus API on May 2, 2019, for the 12-month data and on November 2, 2018, for the 6-month data. These data were also published in our 2019 paper and included here for convenience to readers [35].

### Statistical Analysis

We performed an a priori power calculation to determine the necessary sample size (n=1600 in each arm of the study) to detect differences in our primary outcome of mean citation counts between groups at 12 months. Please see our previously published paper for how we determined the required sample size in each arm of the study [35]. The study was not powered to detect differences in citation counts at 6 or 36 months nor was it powered to detect differences across the subject area–level comparisons at any of the time intervals. To power a study to detect differences at the subject level, each subject would have required between 1000 to 3000 articles in each arm of the study, which was not feasible for us to conduct for budgetary reasons.

Baseline characteristics of articles at the start of the study were tabulated and compared across randomized arms of the study. We categorized articles by subject area, access type (closed vs OA), Journal Impact Factor, and citations and Mendeley saves. Both the primary and secondary outcomes were analyzed with the two-sample *t* test on log-transformed data (1 + x). In the event that mean differences were statistically significant, we calculated effect sizes using Cohen *d* [41] on log-transformed data. Cohen *d* is defined as the difference between two means divided by a SD for the data. Lastly, we performed a stepwise and backward multivariate ordinary least squares (linear) regression analysis to determine the predictors of citations at 6, 12, and 36 months; we used log-transformed dependent variables in the model. A significance value of 0.1 was used for the removal of variables in our stepwise regression model. Journal Impact Factor, access type (ie, OA vs closed), baseline Mendeley saves and citations, and TrendMD clicks (ie, clicks on promoted article links) and impressions (ie, display of promoted article links) were covariates in the regression model; they were selected as covariates because each of them has known independent effects on citations (eg, Journal Impact Factor is a predictor of citation counts) and do not have issues of multicollinearity. We did not collect data on all possible predictors of citations (eg, number of authors or international collaboration) in our regression model, as this was out of scope to our analysis; the primary goal of our regression model was to determine if TrendMD clicks and impressions were independent predictors of our primary outcome of citations at 36 months. All regressions adjusted the SEs for clustering of citations using Huber-White SEs; this corrected for heteroscedasticity [42]. Residuals were analyzed for normality using the Kolmogorov-Smirnov test; we also analyzed the residuals using skewness and kurtosis statistical values and compared to SE values.

A 2-tailed *P*<.05 was considered statistically significant. To mitigate the type I error rate, the Bonferroni correction method was used to control for multiple comparisons made for the 8 disciplinary differences in mean citation counts [43]. A 2-tailed *P*<.006 was considered to be statistically significant for mean differences in Mendeley reader and citation counts across subject areas. Arithmetic mean values for Mendeley saves and citation counts are shown with 95% CIs on non–log-transformed data unless otherwise specified. Tests for normality were included in the model. SPSS version 25 (IBM Corp) was used to complete the statistical analyses.

## Results

### Baseline Characteristics

The following was published verbatim in our previously published randomized controlled trial [35]; these data and discussion are included here for convenience of the reader. Overall, 3200 articles were randomized: 1600 to the TrendMD arm and 1600 to the control arm. The Kolmogorov-Smirnov test of the 6-month Mendeley saves (*P*=.61) and citation count (*P*=.13) data retained the null hypothesis that the distributions were log-normal within the control and TrendMD arms. Tables 1 and 2 show the baseline characteristics of articles randomized to TrendMD versus the control; no statistical tests were used to compare any metrics between groups at baseline according to the CONSORT (Consolidated Standards of Reporting Trials) 2010 guidelines [44].

**Table 1:** Overall baseline characteristics [35].

Intervention	Articles, n	Open access, n	Journal Impact Fact, mean (SD)	Mendeley saves, mean (SD)	Citation count, mean (SD)
Control	1600	88	14.77 (15.87)	23.37 (36.33)	0.86 (2.98)
TrendMD	1600	92	15.41 (16.56)	23.79 (39.34)	1.04 (3.37)

**Table 2:** Baseline characteristics by subject area [35].

Category and intervention		Open access, n	Journal Impact Factor, mean (SD)	Mendeley saves, mean (SD)	Citation count, mean (SD)
**Business, economics, and management**					
	Control	0	5.28 (1.59)	33.99 (43.69)	1.02 (1.60)
	TrendMD	0	5.53 (1.55)	35.38 (35.60)	1.24 (2.26)
**Chemical and materials sciences**					
	Control	1	26.53 (12.20)	23.92 (26.95)	1.53 (7.27)
	TrendMD	1	31.37 (15.89)	25.97 (43.57)	1.58 (6.31)
**Engineering and computer science**					
	Control	3	14.48 (9.74)	10.44 (16.27)	0.29 (0.65)
	TrendMD	2	14.82 (9.93)	10.27 (16.83)	0.35 (0.97)
**Health and medical sciences**					
	Control	11	35.98 (22.48)	37.98 (52.55)	1.93 (2.29)
	TrendMD	7	34.42 (21.87)	38.04 (62.8)	3.1 (5.55)
**Humanities, literature, and arts**					
	Control	2	2.15 (0.86)	13.39 (17.36)	1.06 (2.08)
	TrendMD	2	2.15 (0.87)	13.13 (15.97)	0.95 (2.09)
**Life sciences and earth sciences**					
	Control	52	21.57 (13.8)	43.33 (53.73)	0.37 (0.74)
	TrendMD	51	22.76 (14.42)	44.13 (52.87)	0.48 (0.85)
**Physics and mathematics**					
	Control	10	7.67 (5.86)	8.90 (14.33)	0.33 (1.09)
	TrendMD	11	7.74 (5.85)	9.04 (18.12)	0.31 (1.29)
**Social sciences**					
	Control	9	4.52 (1.08)	15.01 (16.56)	0.36 (1.16)
	TrendMD	18	4.51 (1.06)	14.39 (17.27)	0.35 (1.17)

### Primary Outcome

**Figure 5:**
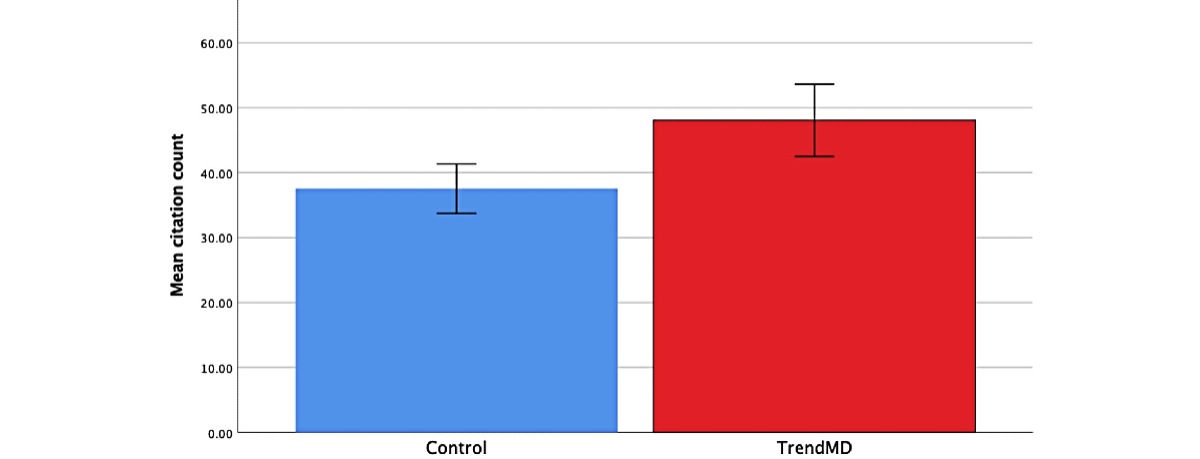
Mean citation count over 36 months: TrendMD versus control.

**Figure 6:**
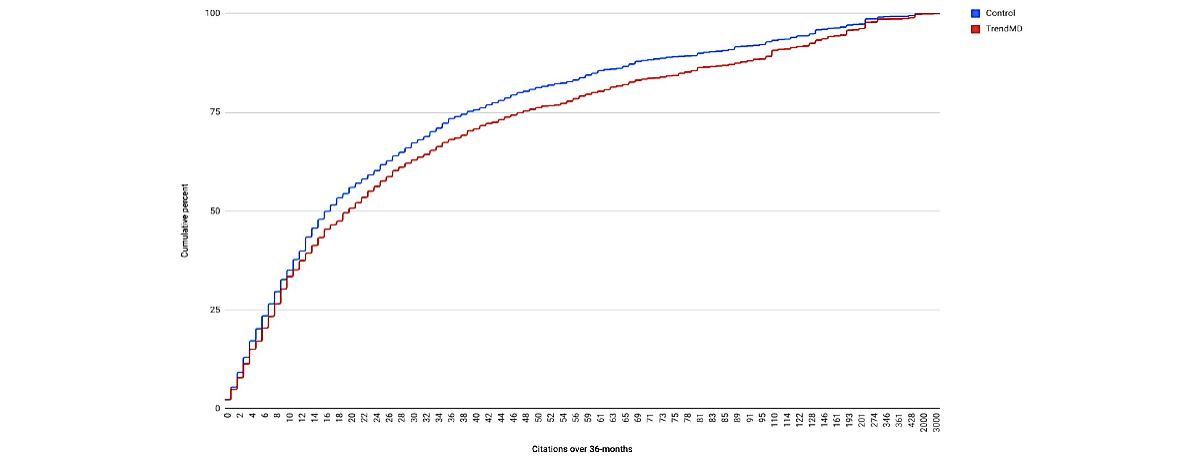
Cumulative distribution of citations over 36 months: TrendMD versus control.

Articles randomized to TrendMD (n=1600) showed a 28% increase in mean citations relative to the control at 36 months (Figure 5). The mean citations for articles randomized to TrendMD was 48.05 (SD 113.51), compared to 37.53 (SD 77.36) for articles randomized to the control. The difference in mean citations for TrendMD articles versus the control was 10.52 (95% CI 3.79-17.25) and was statistically significant (*P*=.001). The effect size of TrendMD on citations at 36 months was small (Cohen *d* 0.11; Table 3). The cumulative distribution of article citations at 36 months is shown in Figure 6. The fact that the TrendMD cumulative distribution curve is shifted to the right relative to the control indicates the promotion of increased citation rates across the entire distribution of articles; the effect was not limited to just a few outlying articles.

**Table 3:** Citation counts at 36, 12, and 6 months.

	36 months		12 months		6 months	
	Control	TrendMD	Control	TrendMD	Control	TrendMD
Citations, mean (SD)	37.53 (77.36)	48.05 (113.51)	10.10 (19.09)	15.16 (40.37)	5.12 (10.65)	6.18 (16.10)
Mean difference in citations (95% CI)	N/A^a^	10.52 (3.79-17.25)	N/A	5.06 (2.87-7.25)	N/A	1.06 (0.12-2.01)
*P* value	N/A	.001	N/A	<.001	N/A	.005
Cohen *d*	N/A	0.11	N/A	0.16	N/A	0.10

^a^N/A: not applicable.

### Secondary Outcomes

#### Subject Area Differences in Mean Citations at 36 Months

**Figure 7:**
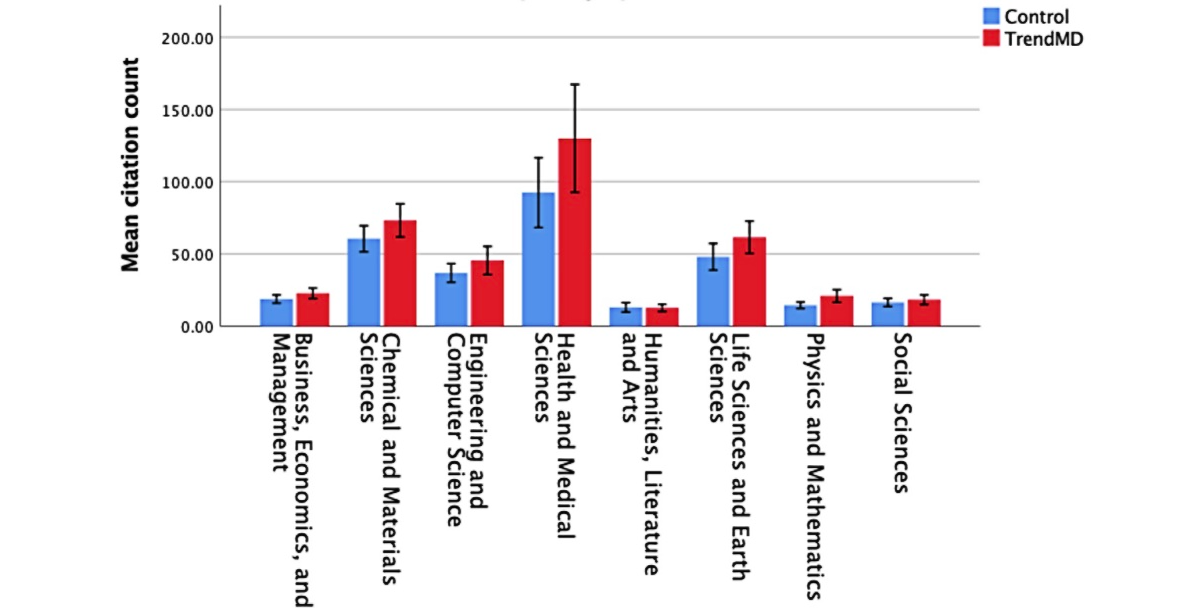
Mean citation counts by subject area over 36 months: TrendMD versus control.

At 36 months, TrendMD was not found to yield statistically significant increases in citation counts relative to the control in any of the individual subject areas. The largest relative difference in citation counts compared to the control was found to be in the subject area of health and medical sciences (41%); however, it was not found to be statistically significant (see Table 4 and Figure 7 for a breakdown of mean differences for each subject area).

**Table 4:** Citations at 36 months by subject area.

Category and intervention		Articles, n		Citations, mean (SD)		Mean difference (95% CI)			*P* value	
**Business, economics, and management**							3.95 (–0.66 to 8.56)			.24
	Control	200	18.75 (20.45)							
	TrendMD	200	22.70 (26.14)							
**Chemical and materials sciences**							12.68 (–1.84 to 27.20)			.18
	Control	200	60.46 (64.48)							
	TrendMD	200	73.14 (82.17)							
**Engineering and computer science**							8.61 (–3.07 to 20.29)			.40
	Control	200	36.80 (46.04)							
	TrendMD	200	45.41 (70.31)							
**Health and medical sciences**							37.49 (–6.91 to 81.89)			.08
	Control	200	92.43 (173.73)							
	TrendMD	200	129.92 (267.99)							
**Humanities, literature, and arts**							–0.35 (–3.73 to 4.43)			.89
	Control	200	12.98 (23.63)							
	TrendMD	200	12.63 (17.40)							
**Life sciences and earth sciences**							13.51 (–0.91 to 27.93)			.03
	Control	200	47.97 (66.14)							
	TrendMD	200	61.48 (79.95)							
**Physics and mathematics**							6.43 (–1.53 to 11.33)			.12
	Control	200	14.46 (16.00)							
	TrendMD	200	20.89 (31.38)							
**Social sciences**							1.90 (–2.4 to 6.20)			.05
	Control	200	16.39 (20.13)							
	TrendMD	200	18.29 (23.52)							

#### Multivariate Regression Analysis

Overall, our multivariate regression models were a good fit to predict citations in all articles at 6, 12, and 36 months. The Kolmogorov-Smirnov test of the residuals (*P*=.21) were normal; values for skewness and kurtosis in the residuals were both less than 1 SE, which suggests that the residual values were not significantly different from the expected value of zero for a normal distribution. Our models predicted 41%, 43%, and 44% of the variation in citation counts at 6, 12, and 36 months, respectively. Clicks driven by TrendMD were found to be an independent predictor of citations; articles that received the greatest number of clicks from TrendMD had the largest citation advantage at 36 months (see Table 5 for the standardized beta coefficients for each covariate in the model).

**Table 5:** Multivariate regression model: standardized beta coefficients at 36, 12, and 6 months.^a^

Standardized beta coefficients	36 months	12 months	6 months
Baseline citations	0.286	0.329	0.323
Baseline Mendeley saves	0.209	0.216	0.163
Journal Impact Factor	0.207	0.175	0.222
Access type	NS^b^ (0.664)^c^	NS (0.847)	NS (0.721)
TrendMD clicks	0.266	0.182	0.153
TrendMD impressions	NS (0.342)	0.063	0.073

^a^All other variables included in the regression model were significant at *P*<.001.

^b^NS: nonsignificant.

^c^Significance values for nonsignificant variables excluded in the model are included in brackets.

## Discussion

To the best of our knowledge, this was the first randomized controlled trial to demonstrate how relatively brief postpublication promotion of peer-reviewed articles over a 6-month period can be used to persistently increase citation rates of those articles after 36 months. The overall citation advantage conferred by article promotion in the TrendMD Network was 28% relative to the control at 36 months, which dissipated from a 50% citation advantage at 12 months. Despite the overall citation advantage observed at 36 months, we did not find a statistically significant increase to citation counts within individual subject areas included in this study. There were, however, larger, albeit nonstatistically significant differences in citation counts measured in certain subjects versus others. For example, health and medical sciences articles saw larger citation increases than articles published in humanities, literature, and arts journals. One possible explanation for this could be because two-thirds of the TrendMD Network are STM journals; therefore, the effect size of promotion is likely to be larger for STM articles rather than humanities-related articles. The other possible explanation for the smaller citation advantages in some subject areas is because of differing publication cycle lengths, which have a direct effect on the speed in which citations accrue overtime. Articles in the fields of medicine, life sciences, and physics, for example, are typically published faster in comparison to articles in the humanities and social sciences [2].

Notwithstanding, our study was not powered to detect differences across the subject area–level comparisons; therefore, the negative findings across individual subject areas are likely due to the small sample sizes and resulting type II errors [45]. Given that we found an overall citation advantage at 36 months and found a Mendeley save advantage in 7 of the 8 subject areas at 6 months, it is reasonable to conclude that studies using larger sample sizes within subject areas over periods longer than 36 months may have shown an increase in citations. Future studies using larger sample sizes within subject areas over longer periods of time are needed to determine whether, and to what degree, promotion of articles in the TrendMD Network confers a citation advantage to individual subject areas.

We initially hypothesized that the overall 50% citation advantage observed at 12 months would increase over time due to the time it takes citations to accrue from manuscripts passing through peer review and onto publication [2]. However, our data suggests the citation advantage conferred to promoted articles dissipated between 12 and 36 months, which could be due to multiple factors affecting article citation rates, such as author credentials, subject matter, and cited references [36]. We do not know the precise timing of the peak of the citation advantage because we only measured citation counts at 6, 12, and 36 months. Future studies are warranted that measure citation rates at more frequent intervals to discern when the citation advantage conferred to promoted articles are at its maximum.

Based on these data, we can speculate on the mechanism in which TrendMD conferred a sustained citation advantage to promoted articles at 36 months. Readers clicking on promoted article links displayed in the widget that they may have not otherwise discovered, saved these articles to their Mendeley reference libraries, and cited these articles while creating their own scholarly work. This pathway is evidenced by the fact that page views (ie, clicks) driven by TrendMD were an independent predictor of both Mendeley saves at 6 months and citations at 6, 12, and 36 months (Table 5).

The findings of our study significantly add to the limited corpus of literature examining the efficacy of strategies to augment citation counts of peer-reviewed research. Though publishing research in OA journals is the most widely studied strategy to augment citation counts, the extent to which the OACA exists or is confounded by selection bias remains unknown [7,11,12,15,16]. In addition, even if the OACA did exist at some point in time in the past, the more that OA content becomes the standard, the less likely the strategy will yield citation benefits as the effect will be ubiquitous across all articles. Other strategies such as the promotion of peer-reviewed literature in social media platforms for the purpose of enhancing citation counts have similarly yielded inconclusive and, at times, conflicting results [21-23,25,26]. These data presented in our study address a pressing unmet need of authors, publishers, and funders for evidence-based strategies that can be used to enhance discoverability by the right targeted audience, which in turn augments the usage and citations counts of peer-reviewed content.

The research presented here has several strengths. First, the outcome of citation counts is unbiased and objective, increasing the reproducibility of the results. Second, we used a rigorous randomized controlled trial design, which minimizes the likelihood of bias and confounding. Third, our sample size was large, and our study was adequately powered for outcomes of differences in mean citations.

There are, however, several limitations to this research. First, all authors have a conflict of interest with the results presented as creators (PK and GE) or employees (TB) of TrendMD. Risk of bias, however, was mitigated by the randomized controlled trial design. Second, although our study was adequately powered to detect mean differences in citation counts across all articles at 12 months, our study was not adequately powered to detect mean differences in citation counts between disciplines. Third, our inclusion criteria of randomly selecting articles published in 8 out of the top 20 journals with the highest h-5 index in Google Scholar categories may make our results less generalizable to articles published in journals with lower Impact Factors. We attempted to mitigate this possible limitation by randomly selecting 8 of the top 20 journals in each subject. Future studies are needed to determine whether TrendMD still confers an enduring citation advantage to articles published in lower Impact Factor journals. Another limitation and question that stems from these data is whether longer periods of promotion beyond 6 months would yield larger and more enduring benefits to citation counts. Future studies using promotion periods longer than 6 months are needed to determine whether there is a dose-dependent relationship between the length of the article promotion period and the magnitude of citation advantage conferred. Our group has previously completed a study showing that there is indeed a dose-dependent relationship between the intensity of article promotion and page views [28]; however, the degree to which the increase in page views leads to a citation advantage is not known. It is also possible that a longer promotion period of articles may saturate over time as readers are presented with the same article links; future studies are needed to determine whether prolonged promotion of articles lead to diminishing rates of return for citations. Lastly, the number and type of publishers participating in the TrendMD Network may change over time, which may affect the reproducibility of our findings [35]. Past studies indicate that the efficacy of TrendMD promotion is dependent on the number and type of publishers participating in the Network [28,29]. In general, replicated data indicates that the more publishers across subject areas using TrendMD the greater the efficacy of the channel to confer benefits to article visibility and usage [35]. If publishers stopped using TrendMD, the channel is unlikely to be as effective at augmenting citation counts as described in this study. More generally, there are, of course, limitations to citation analyses; citations only reflect activity in academia, and the usefulness of citations as indicators varies greatly between fields [46].

Despite the limitations, this study demonstrates that the promotion of articles in a cross-publisher online distribution channel (TrendMD) over a 6-month period can be used to persistently increase citations of articles after 36 months. Though we did not find a statistically significant increase in citation counts within individual subject areas at 36 months, this was likely due to small sample sizes and insufficient power, which resulted in type II errors. The overall citation advantage conferred to promoted articles was observed at 6 months, appeared to peak at 12 months, and endured, albeit to a lower level relative to the control at 36 months.

## Appendix

Multimedia Appendix 1 CONSORT non-eHealth checklist.

## Abbreviations

API: application programming interface
CEO: Chief Executive Officer
CONSORT: Consolidated Standards of Reporting Trials
OA: open access
OACA: open access citation advantage
OCE: Ontario Centres of Excellence
STM: scientific, technical, and medical
